# Epidermal growth factor induces bladder cancer cell proliferation through activation of the androgen receptor

**DOI:** 10.3892/ijo.2012.1593

**Published:** 2012-08-21

**Authors:** KOJI IZUMI, YICHUN ZHENG, YI LI, JACQUELINE ZAENGLE, HIROSHI MIYAMOTO

**Affiliations:** 1 Department of Pathology and Laboratory Medicine, University of Rochester Medical Center, Rochester, NY 14642, USA;; 2 Department of Urology, Second Affiliated Hospital, Zhejiang University School of Medicine, Hangzhou 310009, P.R. China

**Keywords:** androgen receptor, bladder cancer, epidermal growth factor, hydroxyflutamide, Src, transcriptional intermediary factor 2

## Abstract

Androgen receptor (AR) signals have been suggested to contribute to bladder tumorigenesis and cancer progression. Activation of epidermal growth factor receptor (EGFR) also leads to stimulation of bladder tumor growth. However, crosstalk between AR and EGFR pathways in bladder cancer remains uncharacterized. We have recently shown that androgens activate the EGFR pathway in bladder cancer cells. The purpose of this study was to investigate the effects of EGF on AR activity in bladder cancer. EGF increased AR transcriptional activity by 1.2-, 1.9- and 2.0-fold in UMUC3, 5637-AR and J82-AR cell lines, respectively, over mock treatment and a specific EGFR inhibitor, PD168393, antagonized the EGF effect. Combined treatment of EGF and dihydrotestosterone (DHT) further induced AR transactivation while an AR antagonist, hydroxyflutamide (HF), abolished the effect of not only DHT but also EGF. In growth assays, EGF alone/DHT alone/EGF+DHT increased cell numbers by 16/12/19%, 6/14/18% and 30/12/38% in UMUC3-control-shRNA, 5637-AR and J82-AR, respectively, whereas the effects of EGF were marginal or less significant in UMUC3-AR-shRNA (8%) or AR-negative 5637-V (<1%) and J82-V (17%) cells. HF treatment at least partially counteracted the EGF effect on the growth of AR-positive cells. Western blotting demonstrated that EGF, especially in the presence of DHT, upregulated the expression of the p160 coactivator TIF2 and HF again blocked this stimulation. Co-immunoprecipitation revealed the association between AR and estrogen receptor (ER)-β or Src in UMUC3 cells and stronger associations with EGF treatment, implying the involvement of the AR/ER/Src complex in EGF-increased AR transactivation and cell growth. Current results, thus, suggest that EGF promotes bladder cancer cell proliferation via modulation of AR signals. Taken together with our previous findings, crosstalk between EGFR and AR pathways can play an important role in the progression of bladder cancer.

## Introduction

Epidemiological and clinical evidence has indicated a substantially higher risk of urinary bladder cancer in males yet there is a tendency showing more aggressive behavior in tumors from female patients (1,2). Recent experimental data suggest that urothelial carcinoma, like prostate and breast cancers, is an endocrine-related neoplasm (reviewed in ref. 3). In particular, the androgen receptor (AR) and estrogen receptor (ER) signaling pathways have been shown to contribute to bladder tumorigenesis and cancer progression (3–13), which may explain some of the differences in male versus female bladder cancer.

Activation of the epidermal growth factor (EGF) receptor (EGFR) family is known to involve the growth and progression of a variety of malignancies. In bladder cancer, EGFR/ERBB2 is frequently overexpressed, which correlates with higher tumor grade/stage and poorer prognosis (14–16). Experimental evidence in bladder cancer has also suggested that the EGFR pathway plays a critical role in cell proliferation, apoptosis, differentiation, migration and angiogenesis (17–19). Consequently, the efficacy of targeted therapy directed at EGFR signals has been assessed in bladder cancer.

The crosstalk between nuclear hormone receptors and growth factors leads to activation of nuclear receptor-mediated transcription. Specifically, in prostate cancer cells, AR signals upregulate *EGFR* and *ERBB2* gene expression, whereas activation of EGFR and ERBB2 modulates AR functions (20–24). It has also been shown that the assembly of the EGFR/AR/ER/Src signaling complex is crucial for proliferation of prostate and breast cancer cells triggered by androgens, estrogens and/or EGF (25). In contrast, the relationship between the AR and EGFR pathways in bladder cancer remains poorly understood. We have recently shown that AR activation results in upregulation of EGFR and ERBB2 expression in bladder cancer cells, which may play an important role in androgen-mediated tumor progression (26). In the present study, we investigated whether EGF could alter AR activity in bladder cancer cells.

## Materials and methods

### Cell culture and chemicals

Human bladder cancer cell lines, UMUC3, 5637 and J82, obtained from the American Type Culture Collection (Manassas, VA, USA) were maintained in Dulbecco’s modified Eagle’s medium (Mediatech, Manassas, VA, USA) supplemented with 10% fetal bovine serum (FBS) at 37°C in a humidified atmosphere of 5% CO_2_. Cells were cultured in phenol-red free medium supplemented with 5% charcoal-stripped FBS at least 18 h before experimental treatment. We obtained dihydrotestosterone (DHT) and EGF from Sigma (St. Louis, MO, USA); hydroxyflutamide (HF) from Schering (Kenilworth, NJ, USA); and PD168393 from Calbiochem (San Diego, CA, USA).

### Stable cell lines with AR and AR-short hairpin RNA (shRNA)

Cell lines stably expressing a full-length wild-type human AR (5637-AR and J82-AR) or vector only (5637-V and J82-V) were established, using a lentivirus vector (pWPI-AR or pWPI-control) with psPAX2 envelope and pMD2.G packaging plasmids, as we described previously (11,26). Similarly, stable AR knockdown/control cell lines (UMUC3-AR-shRNA/UMUC3-control-shRNA) were established with a retrovirus vector pMSCV/U6-AR-shRNA or pMSCV/U6-control-shRNA (5,26).

### Reporter gene assay

Bladder cancer cells at a density of 50–60% confluence in 24-well plates were co-transfected with 250 ng of MMTV-luc reporter plasmid DNA and 2.5 ng of pRL-TK-luc plasmid DNA, using GeneJuice transfection reagent (Novagen, Gibbstown, NJ, USA). Six hours after transfection, the medium was replaced with one supplemented with 5% charcoal-stripped FBS containing ethanol or ligands (DHT, HF, EGF and/or PD168393) for 24 h. Cells were harvested, lysed and assayed for luciferase activity determined using a dual-luciferase reporter assay kit (Promega, Madison, WI, USA) and luminometer (TD-20/20; Turner BioSystems, Sunnyvale, CA, USA).

### Cell proliferation assay

We used the MTT (methyl thiazolyl diphenyl tetrazolium bromide) assay to assess cell viability, as described previously (26,27). Briefly, cells (3×10^3^) seeded in 96-well tissue culture plates were incubated with medium supplemented with charcoal-stripped FBS in the presence or absence of ligands (DHT, HF and EGF). The media were refreshed every 24 h. After 96 h of treatment, 10 *μ*l MTT (Sigma) stock solution (5 mg/ml) was added to each well with 0.1 ml of medium for 4 h at 37°C. The medium was replaced with 100 *μ*l DMSO followed by incubation for 5 min at room temperature. The absorbance was then measured at a wavelength of 570 nm with background subtraction at 655 nm.

### Western blotting

Protein extraction and western blotting were performed, as described previously (27) with minor modifications. Briefly, equal amounts of protein (20 *μ*g) obtained from cell extracts were separated in a 10% sodium dodecylsulfate (SDS)-polyacrylamide gel electrophoresis (PAGE) and transferred to polyvinylidene difluoride membrane (Millipore, Billerica, MA, USA) by electroblotting using a standard protocol. Specific antibody binding was detected, using an anti-AR antibody (clone N20; diluted 1:2,000; Santa Cruz Biotechnology, Santa Cruz, CA, USA), an anti-transcriptional intermediary factor 2 (TIF2) antibody (clone 29/TIF2; diluted 1:1,000; BD Bioscience, Franklin Lakes, NJ, USA), or an anti-GAPDH antibody (clone 6C5; diluted 1:1,000; Santa Cruz Biotechnology), with horseradish peroxidase detection system (SuperSignal West Pico Chemiluminescent Substrate; Thermo Scientific, Rockford, IL, USA).

### Co-immunoprecipitation

UMUC3 cells were treated with mock (ethanol) or EGF for 24 h and protein (500 *μ*g) from the cell lysates was incubated with 2 *μ*g of anti-AR antibody (N20) or normal rabbit IgG (Santa Cruz Biotechnology) for 16 h at 4°C with agitation. To each sample we added 20 *μ*l of protein A/G-agarose beads (Santa Cruz Biotechnology), incubated for 1 h and washed thrice with radio-immunoprecipitation assay buffer. Then, the complex was resolved on a 10% SDS-PAGE, transferred to the membrane and blotted with an anti-ERβ antibody (clone 14C8; diluted 1:500; Abcam, Cambridge, MA, USA) or an anti-v-Src antibody (clone 327; diluted 1:1,000; Calbiochem).

### Statistical analysis

Student’s t-test was used to analyze differences in relative luciferase activity and relative cell number between the two groups. P<0.05 was considered statistically significant.

## Results

### EGF mediates AR transactivation via EGFR

**Figure 1.**
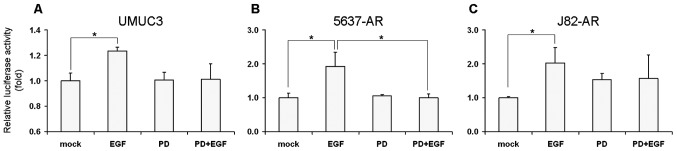
Effects of EGF on AR transactivation. Bladder cancer cells (A, UMUC3; B, 5637-AR; C, J82-AR) were transfected with MMTV-Luc and were then cultured for 24 h in the presence of ethanol (mock), 100 ng/ml EGF and/or 1 *μ*M PD168393, as indicated. Luciferase activity analyzed in a luminometer is presented relative to that of mock treatment in each cell line (first lanes; set as 1-fold). Each value represents the mean + SD from at least three independent experiments. ^*^p<0.05.

Because previous studies showed ligand-independent activation of AR transcription by EGF in prostate cancer cells (20–22), we first assessed the effects of EGF and a specific EGFR inhibitor PD168393 on AR transactivation in bladder cancer lines. In AR-positive UMUC3 and AR-negative 5637 and J82 with a full-length AR stably expressed by lentivirus, luciferase activity was determined in the cell extracts with transfection of a plasmid (MMTV-luc) containing an androgen response element (ARE) as a reporter of AR-mediated transcriptional activity. As shown in Fig. 1, EGF treatment increased luciferase activity by 1.2-, 1.9- and 2.0-fold in UMUC3 (p=0.013), 5637-AR (p=0.036) and J82-AR (p=0.050), respectively, over mock treatment. PD168393 showing only marginal activity (in UMUC3 and 5637-AR) or some agonist effect (1.5-fold in J82-AR) could antagonize the EGF effect on AR transcription. In AR-knockdown UMUC3-AR-shRNA and AR-negative lines (5637, 5637-V, J82 and J82-V), EGF and/or PD168393 showed marginal effects on AR transcriptional activity (data not shown). These results suggest that EGF induces AR transactivation via EGFR in an androgen-independent manner.

### Antiandrogen blocks EGF-induced AR transactivation

**Figure 2.**
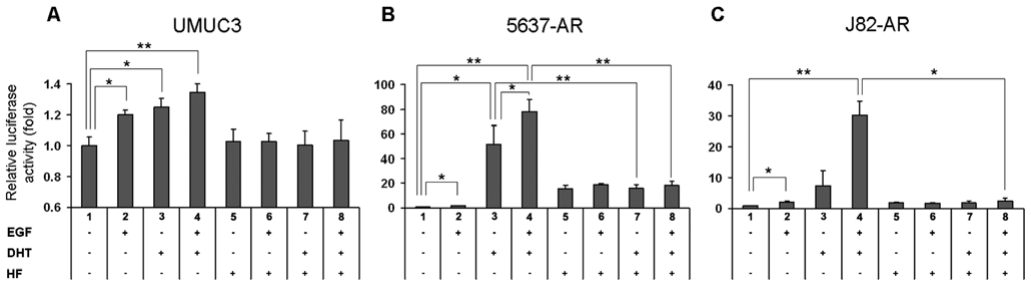
Effects of androgen and antiandrogen on EGF-mediated AR transactivation. Bladder cancer cells (A, UMUC3; B, 5637-AR; C, J82-AR) were transfected with MMTV-Luc and were then cultured for 24 h in the presence of ethanol (mock), 100 ng/ml EGF, 10 nM DHT and/or 10 *μ*M HF, as indicated. Luciferase activity analyzed in a luminometer is presented relative to that of mock treatment in each cell line (first lanes; set as 1-fold). Each value represents the mean + SD from at least three independent experiments. ^*^p<0.05; ^**^p<0.01.

We next assessed the effect of EGF, in conjunction with androgen and/or antiandrogen, on AR transcriptional activity in bladder cancer cells. As shown in Fig. 2A, DHT treatment increased AR transcription by 25% (lanes 1 vs. 3, p=0.032) and addition of EGF further induced it by 35% (lanes 1 vs. 4, p=0.001; lanes 3 vs. 4, p=0.103) in UMUC3. Interestingly, HF showing only marginal activity (lanes 1 vs. 5) abolished the effects of not only DHT (lanes 3 vs. 7, p=0.077) but also EGF (lanes 2 vs. 6, p=0.061) and EGF+DHT (lanes 4 vs. 8, p=0.082). Similarly, in 5637-AR (Fig. 2B) and J82-AR (Fig. 2C), DHT (lane 3) induced AR transcription to 52- and 7.4-fold, respectively and EGF in the presence of DHT (lanes 4 vs. 3) enhanced it to 78- (p= 0.035) and 30-fold (p=0.054), respectively. HF showing some agonist activities (lanes 1 vs. 5) in 5637-AR (15-fold)/J82-AR (1.8-fold), which were much higher (vs. 1.7-fold)/similar (vs. 2.1-fold) compared to EGF stimulations (lane 2), could block the effects of DHT (lanes 3 vs. 7, p=0.005/p=0.164) and EGF+DHT (lanes 4 vs. 8, p=0.009/p=0.013). Again, in UMUC3-AR-shRNA, 5637(-V) and J82(-V) cells, EGF, DHT and/or HF showed marginal effects on AR transcription (data not shown). These findings suggest that EGF and androgen cooperatively induce AR transactivation that is sufficiently inhibited by an anti-androgen.

### EGF stimulates cell growth via AR signaling

**Figure 3.**
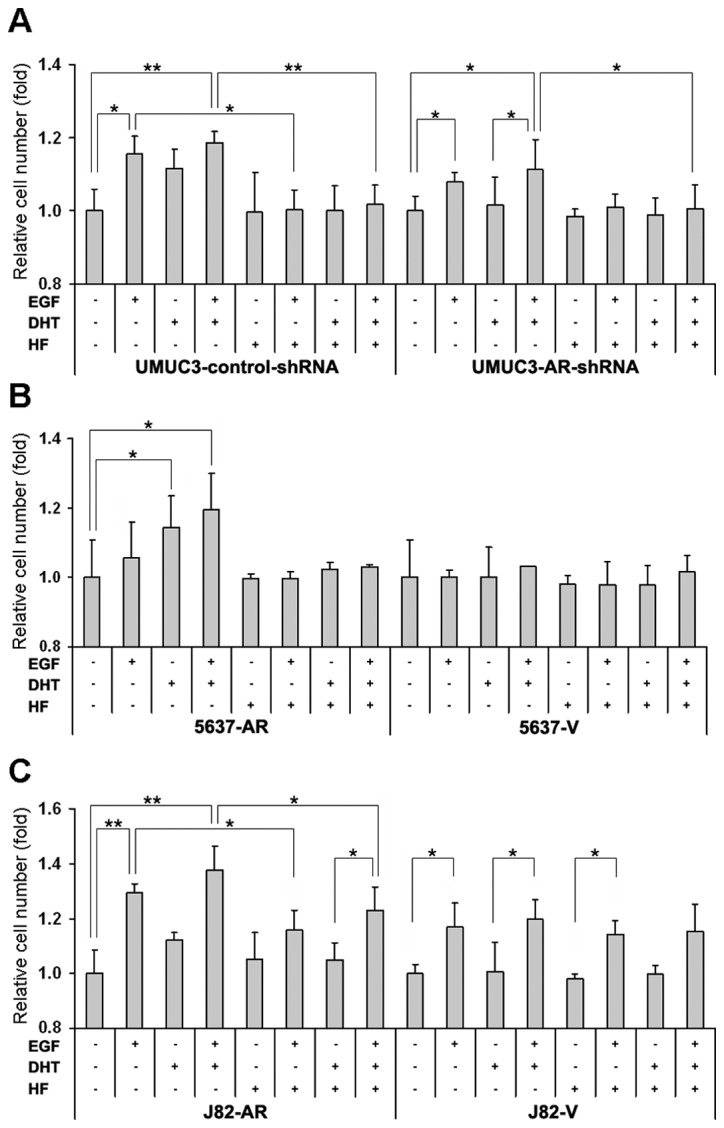
Effects of EGF on cell viability. Bladder cancer cells (A, UMUC3-control-shRNA/AR-shRNA; B, 5637-AR/vector; C, J82-AR/vector) were cultured for 4 days in the presence of ethanol (mock), 100 ng/ml EGF, 10 nM DHT and/or 10 *μ*M HF, as indicated. Cell viability was assayed with MTT and growth induction is presented relative to cell number with mock treatment estimated by measuring the absorbance at a wavelength of 570 nm with a background subtraction at 655 nm (first lanes; set as 1-fold). Each value represents the mean + SD from at least three independent experiments. ^*^p<0.05; ^**^p<0.01.

We then performed the MTT assay to investigate the effects of EGF androgen and antiandrogen on cell proliferation of bladder cancer lines with vs. without AR (i.e., UMUC3-control-shRNA vs. UMUC3-AR-shRNA, 5637-AR vs. 5637-V and J82-AR vs. J82-V). As shown in Fig. 3A, in UMUC3-control-shRNA, treatment of EGF, DHT and EGF+DHT increased cell growth by 16% (p=0.020), 12% (p=0.195) and 19% (p=0.009), respectively, over mock treatment and HF treatment appeared to restore the growth to the basal levels. In UMUC3-AR-shRNA, DHT effect was marginal (2%) and the effects of EGF (8%, p=0.039) and EGF+DHT (11%, p=0.040) were less significant compared to those in UMUC3-control-shRNA. In 5637-AR, treatment of EGF, DHT and EGF+DHT induced cell growth by 6% (p= 0.558), 14% (p=0.016) and 19% (p=0.050), respectively and HF almost completely abolished the stimulation (Fig. 3B). In 5637-V, only marginal effects of EGF, DHT and/or HF on cell numbers were seen. In J82-AR, treatment of EGF, DHT and EGF+DHT induced cell growth by 30% (p=0.001), 12% (p=0.179) and 38% (p<0.001), respectively (Fig. 3C). Interestingly, HF was able to antagonize the DHT effect but only partially blocked the EGF effect. As expected, in J82-V, DHT did not increase cell growth, while EGF and EGF+DHT, although less significant, induced it by 17% (p=0.010) and 20% (p=0.043), respectively. Additionally, HF failed to block the EGF effect in J82-V cells. These results suggest that EGF promotes bladder cancer cell proliferation at least partially through the AR pathway.

### EGF increases AR and TIF2 expression

**Figure 4.**
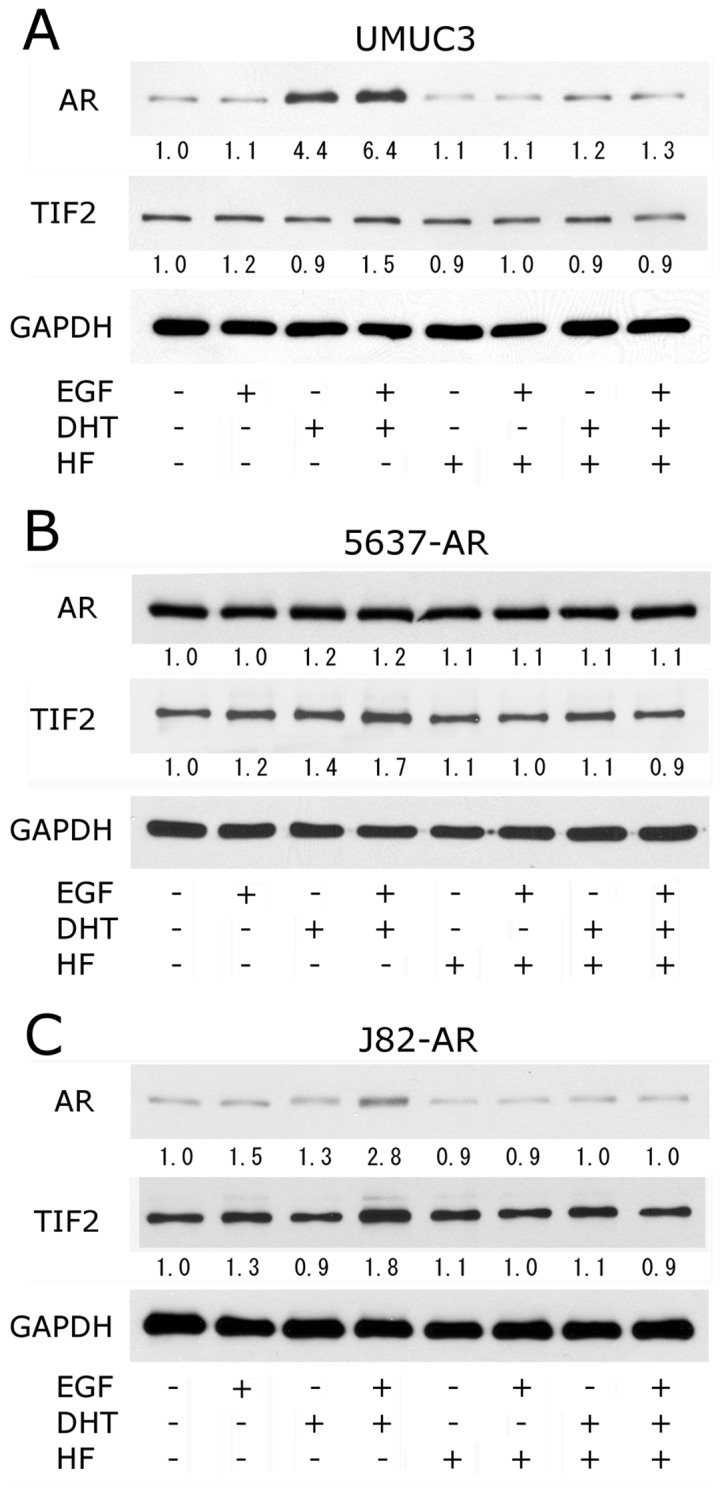
Effects of EGF on AR and TIF2 protein expression. Bladder cancer cells (A, UMUC3; B, 5637-AR; C, J82-AR) were cultured for 24 h in the presence of ethanol (mock), 100 ng/ml EGF, 10 nM DHT and/or 10 *μ*M HF, as indicated. Equal amounts of protein extracted from each cell line were immunoblotted for AR (110 kDa, upper), TIF2 (160 kDa, middle), or GAPDH (37 kDa, lower) as indicated. Densitometry values for specific bands standardized by GAPDH that are relative to those of mock treatment (first lanes; set as 1-fold) are included below the lanes.

To further investigate how EGF influences AR signals, we examined AR expression by western blotting. In UMUC3, AR expression was increased by DHT (4.4-fold) and further enhanced by addition of EGF (6.4-fold), whereas no significant effect of EGF or HF was seen in the absence of DHT (Fig. 4A). HF clearly antagonized the effects of DHT with or without EGF. In J82-AR, EGF appeared to increase AR expression both in the presence (2.8-fold) and absence (1.5-fold) of DHT and HF abolished these effects (Fig. 4C). In contrast, only marginal effects of EGF and/or DHT on AR expression were observed in 5637-AR (Fig. 4B).

Because EGF was shown to induce AR transcription by upregulating TIF2 expression in prostate cancer cells (21), we then determined the levels of TIF2 expression in bladder cancer cell lines upon treatment with EGF, androgen and/or antiandrogen. As shown in middle panels of Fig. 4, EGF increased TIF2 expression in the presence (1.5- to 1.8-fold) and absence (1.2- to 1.3-fold) of DHT. DHT alone increased TIF2 expression only in 5637-AR (1.4-fold) and showed marginal effects in UMUC3 and J82-AR. In addition, HF abrogated EGF- and/or DHT-enhanced TIF2 expression in all these three lines.

### EGF induces AR association with ER and Src

**Figure 5.**
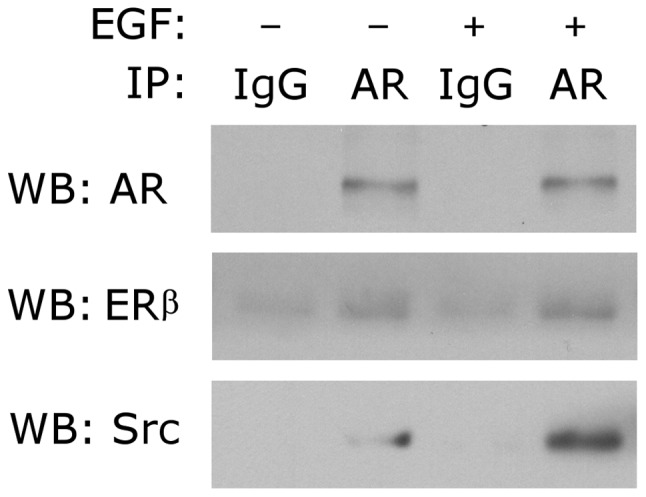
Effects of EGF on AR/ER/Src association. UMUC3 cells were cultured for 24 h in the presence of ethanol (mock) or 100 ng/ml EGF. Cell lysates were immunoprecipitated with anti-AR antibody or normal rabbit IgG and were then immunoblotted for AR (110 kDa), ERβ (56 kDa), or Src (60 kDa), as indicated.

Previous studies in prostate and breast cancers demonstrated that EGF induced AR/ER/Src association, resulting in activation of Src signaling (25,28) and that Src signals phosphorylated tyrosine residue of AR, provoking its transactivation and cell proliferation (29). We therefore investigated whether EGF induced AR/ER/Src complex formation in UMUC3 which is ERα-negative/ERβ-positive (figure not shown). As shown in Fig. 5, both Src and ERβ were co-immunoprecipitated with AR in bladder cancer cells. Furthermore, EGF treatment facilitated the association of AR with ERβ or Src.

## Discussion

Dysregulation of the EGFR family is well known to associate with bladder cancer (14–16). AR signals have also been implicated in bladder carcinogenesis and tumor progression (3,5,7,9–13). Nonetheless, crosstalk between the AR and EGFR pathways remains unclear in bladder cancer, although it has been widely studied in prostate cancer (20–24). We have recently shown that AR signals increase EGFR and ERBB2 expression and activity, suggesting androgen-mediated bladder cancer progression via the regulation of the EGFR/ERBB2 pathways (26). In the present study, we provided evidence suggesting that EGF could regulate cell proliferation by activating AR signals in bladder cancer.

In prostate cancer, accumulating evidence has indicated that EGFR/ERBB2 signals induce AR transactivation in an androgen-dependent and -independent manner (20–22). In bladder cancer cells, we here showed that EGF could activate AR transcription and PD168393, a specific inhibitor of EGFR, restored this EGF effect. These data suggest that EGF androgen-independently induces EGFR-mediated ARE reporter activity in bladder cancer. However, it was shown that the effect of EGF on AR transcription might be almost negligible compared to the induction by androgens in prostate cancer (20,21). Similarly, in bladder cancer lines 5637-AR and J82-AR where a wild-type AR was stably overexpressed, the effect of EGF was less significant than that of DHT. On the other hand, in UMUC3 cells that possess endogenous AR, EGF effect (20% increase) is similar to the relatively insignificant effect of DHT (25% increase). In addition, PD168393 displayed agonist effects [1.5-fold (vs. 2.0-fold by EGF or 7.4-fold by DHT)] on AR transcription in J82-AR via unknown mechanisms. It was described in prostate cancer cells that PD168393 upregulated AR target gene expression in the presence of androgen, possibly via blocking basal activity of EGFR or ERBB2 (30). Importantly, as shown in prostate cancer (21), a combination of EGF and androgen further induced AR transcriptional activity in all the three bladder cancer lines tested and the AR antagonist HF completely abolished AR transactivation induced by EGF, androgen, or both at least in UMUC3. We could not evaluate antagonistic effects of HF on EGF-induced AR transcription due to the considerable agonist activity of HF which was even higher than that of EGF in 5637-AR. Thus, our results support the possibility that EGF mediates AR transcriptional activity through the EGFR and AR pathways in bladder cancer cells.

Consistent with previous findings shown by others and us (5,7,9,26) androgens promoted AR-positive bladder cancer cell proliferation that was blocked by antiandrogens. These effects of androgens were suggested to be at least partially mediated through the EGFR pathway (26). In the present study, as expected, EGF increased the growth of AR-positive cells and, less significantly, that of AR-knockdown/negative cells. In AR-positive lines, combined treatment with EGF and androgen further induced cell proliferation. Of note were inhibitory effects of the AR antagonist on EGF- and EGF+androgen-increased cell growth. Specifically, on the growth of 5637-derived lines, EGF and/or DHT showed only marginal effects (5637-V) and HF almost completely abolished EGF-mediated effects (5637-AR). These findings indicate that EGF-induced cell proliferation involves the AR pathway in bladder cancer. Nonetheless, in J82-derived lines, EGF retained its effect on cell growth without AR (J82-V) and HF failed to completely inhibit EGF-increased cell proliferation (J82-AR), suggesting the involvement of those other than the AR pathway.

It has been reported that EGF is capable of inducing AR transcription and protein expression in androgen-independent prostate cancer cells (21). Others also described negative regulation of AR expression and activity by EGFR signaling in prostate cancer (30,31). In bladder cancer cells, we previously showed increases in the expression of endogenous AR by androgen treatment (26), which was inconsistent with the results demonstrated by Boorjian *et al*(9). We also showed no significant increases in exogenously overexpressed AR (5637-AR) by DHT or in endogenous and exogenous ARs by EGF (26). We confirmed our previous findings in the three lines tested and further showed EGF-enhanced AR overexpression in the presence of androgen in UMUC3 and J82-AR, but not in 5637-AR. The mechanism underlying this discrepancy in the response to the treatment of EGF+DHT between levels of exogenous AR expression in 5637 versus J82 remains uncertain. Repeatedly, the AR expression increased by androgen with or without EGF in bladder cancer cells was abolished by an AR antagonist.

EGF has been shown to enhance the expression or phosphorylation of TIF2, one of the p160 nuclear receptor coactivators, leading to an increase in AR transactivation in prostate cancer cells (21). Indeed, the expression of major AR coactivators, including TIF2, was detected in bladder cancer cell lines as well as in AR-positive and even AR-negative bladder tumor specimens and TIF2 knockdown resulted in a decrease in androgen-mediated cell proliferation (9). We here found that TIF2 was considerably (e.g., ≥1.5-fold) augmented in the presence of EGF and DHT in bladder cancer cells, while EGF or DHT alone could lead to marginal/only slight increases in TIF2 expression. Interestingly, like our results in AR expression/activity and cell proliferation, EGF-induced TIF2 upregulation was abolished by the antiandrogen. Although detailed mechanisms need to be clarified, these results may imply that elevated levels of TIF2 contribute to EGF/androgen-enhanced AR trans-activation in bladder cancer cells.

In hormone-responsive cells expressing both AR and ER (α and/or β), such as prostate and breast cancers, AR/ER/Src association plays a crucial role in activation of Src signals triggered by EGF and/or sex hormones (25,28). It was noteworthy that either AR or ER antagonist sufficiently inhibited this EGF-mediated association and subsequent stimulatory effects (28). It has also been shown that Src mediates EGF-induced AR tyrosine phosphorylation in prostate cancer cells, which leads to an increase in AR transcriptional activity (29). Indeed, in many bladder cancer tissue specimens, AR and ER(s) were found to be co-expressed (3,10,12). In this study, we showed associations of AR with ERβ and Src in UMUC3 which were enhanced by EGF treatment. These findings suggest that EGF activates Src via assembling the AR/ER/Src complex, resulting in AR transactivation and cell proliferation in bladder cancer. This may also justify the drastic inhibition of EGF-induced effects accomplished by antiandrogen treatment.

In conclusion, EGF could increase AR transcriptional activity and cell proliferation in bladder cancer. These EGF effects were likely mediated through the AR pathway involving upregulation of TIF2 expression as well as activation of Src signals due to forming an AR/ER/Src complex. These results, together with our previous findings, not only shed light on crosstalk between the AR and EGFR pathways in bladder cancer but also enhance the feasibility of androgen deprivation interfering with this crosstalk as a potential therapeutic approach.

## Abbreviations:

AR: androgen receptor;
ER: estrogen receptor;
EGF: epidermal growth factor;
EGFR: epidermal growth factor receptor;
FBS: fetal bovine serum;
DHT: dihydrotestosterone;
HF: hydroxyflutamide;
shRNA: short hairpin RNA;
SDS: sodium dodecylsulfate;
PAGE: polyacrylamide gel electrophoresis;
TIF2: transcriptional intermediary factor 2;
ARE: androgen response element
